# Viral topic about the COVID-19 vaccination: the attitudes towards it and the relationship with the well-being and religiosity in a group of Polish students

**DOI:** 10.1192/j.eurpsy.2022.1262

**Published:** 2022-09-01

**Authors:** K. Karakula, A. Forma, E. Sitarz, J. Rog, J. Baj, D. Juchnowicz, H. Karakula-Juchnowicz

**Affiliations:** 1 Medical University of Lublin, 1st Department Of Psychiatry, Psychotherapy And Early Intervention, Lublin, Poland; 2 Medical University of Lublin, Department Of Forensic Medicine, Lublin, Poland; 3 Medical University of Lublin, Chair And Department Of Anatomy, Lublin, Poland; 4 Medical University of Lublin, Department Of Psychiatric Nursing, Lublin, Poland

**Keywords:** mental health, religiosity, Covid-19, vaccination

## Abstract

**Introduction:**

The COVID-19 pandemic currently remains the most significant stressor affecting the global population. Researchers continually report widespread mistrust and negative attitudes towards vaccination, but only a little focus on its association with the emotional well-being.

**Objectives:**

We aimed to investigate the attitudes towards vaccination against COVID-19, as well as its relationship with well-being and religiosity after one year of the pandemic duration amongst Polish students.

**Methods:**

We conducted an anonymous online cross-sectional survey between 12^th^ April – 1^st^ June 2021 amongst Polish students (n=1202). To evaluate emotional distress, we used the Depression, Anxiety, and Stress Scale-21 (DASS-21), for measuring spirituality/religiosity we used The Duke University Religion Index.

**Results:**

The highest rate of vaccinated individuals was noted in a group of medical students (69.9%), the lowest - among responders studying science (1.9%). Students who wanted to be vaccinated had higher levels of depressive, anxiety, and stress symptoms compared to those who were already vaccinated (*p*=0.04); they also had higher depressive symptoms than unvaccinated and unwilling participants (*p*=0.028). Students who didn’t want to be vaccinated against COVID-19 showed the highest religiosity compared to those who would like to be vaccinated (*p*<0.001) or were vaccinated (*p*=0.003). There was a negative correlation between the level of religiosity and severity of depressive and anxiety symptoms (*p*=0.002).

**Conclusions:**

1. The attitudes towards vaccination against COVID-19 depended on the fields of study. 2. Religiousness has been linked with the attitudes towards COVID-19 vaccination as well as level of depression and anxiety amongst Polish students.

**Disclosure:**

No significant relationships.

